# Analysis of microbiological profiles of Indian patients with peri-implantitis and periodontitis

**DOI:** 10.6026/973206300200615

**Published:** 2024-06-30

**Authors:** Sachin B. Mangalekar, Maliha Sultana, Abhishek Mulay, Haripriya Vaddalapu, Devashri P. Newaskar, Shraddanand Bacha, Pranav V Manek, Prachi Desai

**Affiliations:** 1 Department of Periodontology, Bharati Vidyapeeth (Deemed to be University) Dental College and Hospital, Sangli, Maharashtra, India; 2 Al Badar Rural Dental College and Hospital Affiliated to Rajiv Gandhi University of Health Sciences Karnataka, India; 3 Department of Oral and Maxillofacial Surgery, Yashwantrao Chavan Dental College and Hospital, Ahemdnagar, Maharashtra, India; 4 MNR Dental College & Hospital, Affiliated to KNR University of Health Sciences, Telangana, India; 5 Dr. Motadus Multi-Speciality Dental clinic, Implant and Maxillofacial Rehabilitation Centre, Pune - 411001, India; 6 Department of Periodontics, S.B.Patil Institute for Dental Science, Bidar, Karnataka, India; 7 Department of Oral Medicine and Radiology, Pacific Dental College and Research Centre, Udaipur, Rajasthan, India; 8 Oral Pathology and Microbiology, Ahmedabad Dental College and Hospital, Ahmedabad, India

**Keywords:** Microbial profile, periimplantitis, healthy subjects, periodontitis

## Abstract

The microbial profile of patients with periimplantis to inqure any unique composition of microorganisms in them is of interest to dentists. Hence, we evaluated the microbial profile of patients with peri-implantitis, patients with periodontitis and normal healthy subjects. 180 subjects were included in this study. Plaque samples were collected from 60periodontically healthy (PH) participants, 60 periodontitis (PT) subjects, and 60 periimplantitis (PI) subjects. Final concentrations were obtained for seven most common periodontal pathogens namely *Aggregatibacter actinomycetemcomitans*, *Fusobacterium nucleatum*, *Treponema denticola*, *Porphyromonas gingivalis*, *Prevotella intermedia*, *Staphylococcus aureus* and *Haemophilus parainfluenzae*. The abundance of microorganisms was represented in the form of Log10CFU (x103). The abundance of periopathogens evaluated in this research was different in periimplantitis patients, periodontitis patients and normal healthy subjects with slight greater abundance of peripathogens in patient with periimplantitis.

## Background:

Restorations supported by implants are regarded as a necessary procedure in contemporary prosthetic dentistry [1, 2-3]. However, there have been reports of biological as well as technical issues related to dental implant rehabilitation. After a few years of use, biological issues related to implants, such as peri-implant mucositis and periimplantitis, are quite frequently noticed [2-4]. The average incidence of peri-implantitis is believed to be observed to vary between 8.9 and 43.3% at the implant location throughout a 10-year monitoring period, dependant on indicators of risk and risk variables [3, 5- 6].Durable dental implants now have an average survival rate of over 96% thanks to advancements in prosthodontic as well as surgical methods [7, 8- 9]. However, as more patients receive implants on an annual basis, the number of cases of peri-implant ailments has steadily grown; as of late, it has been estimated to affect forty-five percent of patients [10, 11-12]. Following preliminary bone remodeling, periimplantitis (PI) is a degenerative peri-implant disease characterized by an inflammatory infection in the peri-implant tissues and subsequent loss of the bone that supports the implant [13, 14- 15]. Risk factors for periodontitis (PT) include inadequate brushing and flossing, previous experiences of severe PT, and the absence of routine maintenance care [16, 17- 18]. Most remarkably, it has been established that individuals with a previous diagnosis of periodontitis are more likely to develop peri-implantitis [12-14].P articularly, bacteria in the biofilm of implants and teeth release toxins that negatively impact tissue, exacerbate the response to inflammation in the host, and eventually trigger the adjacent tissue to be destroyed [14- 17]. Oral infection with polymicrobial species is linked to both PT and PI, which are inflammatory disorders [19, 20, 21, 22-23]. But the tissues in vicinity of dental implants differ greatly from the tissues adjacent to teeth in several ways, such as the lack of the periodontal ligament, the poorly developed vascular system, and the configuration of the connective tissues [24, 25, 26-27]. These variations increase the likelihood that PI will deteriorate and quickly expand to supporting bone [11-14]. Previous research has demonstrated that the microbiota around periodontal affected teeth and failed implants produce compositions that are comparable, with a significant amount of gram-negative anaerobic rods [13-17]. A number of research investigations have found high amounts of *S. aureus* inside deeper peri-implant periodontal pockets that tend to be suppurative and exhibit bleeding upon probing, along with the bacteria that are frequently suspected of causing periodontal disorders. Notable is the fact that titanium coatings are known to have a particular attraction for *S. aureus* [14-16]. Evidence of transfer from subgingival tooth areas to peri-implant niches has been observed in microbiological analyses. Moreover, it has been demonstrated that the subgingival biofilm composition of implant regions linked to normal good healthy dental implants differs significantly from that of pathogenic or deteriorating implant sites [13-19]. Knowing the features of the microbiota linked to PI may aid in the development of effective preventative measures and therapeutic approaches unique to PI [20-24]. Therefore, it is of interest to evaluate the microbial profile of patients with periimplantitis, patients with periodontitis and normal healthy subjects.

## Methods and materials:

## Study population and clinical examination:

180 subjects were included in this study. Plaque samples were collected from 60 PH participants, 60PT subjects, and 60 PI subjects (Table 1).

## The following were the inclusion criteria:

[1] Participants having at least twenty teeth

[2] The patient's medical history shows no signs of systemic disorders that could impact periodontal health

[3] No periodontal therapy in the previous three months

[4] Participant has not used anti-inflammatory or systemic antibiotics in the previous six months.

## The following were the exclusion criteria:

[1] Participants either nursing or pregnant

[2] Participants either a persistent oral mucosal lesion or an acute infection

[3] Participants who were heavy smoker (more than 20 cigarettes a day).

One periodontist performed full-mouth clinical examinations to assess the state of the peri-implant and periodontal tissues. The gingival index (GI), plaque index (PI), clinical attachment level (CAL) and probing pocket depth (PPD) were assessed during these exams. When participants had no radiological bone loss, no clinical attachment loss, bleeding on probing ≤10% and PPD ≤3 mm they had been considered to have normal periodontal health. The diagnosis for PT was either 1) clinical attachment loss in the interdental area is evident at ≥2 non-adjacent teeth, or 2) clinical attachment loss in the buccal area of the mouth is detectable at ≥2 teeth with PPD >3 mm. Subjects with implants showing radiographic evidence of PPD ≥6 mm and/or marginal bone loss ≥3 mm together with extensive bleeding were diagnosed with PI. The length of the distance from an established landmark (the implant-abutment intersection) to the bottom of the implant sulcus or pocket was used to calculate the CAL of the implant [25]. There were no unhealthy implants in the PT or PH groups, and each participant was allocated to just one group.

## Plaque specimen collection:

During the complete oral cavity periodontal assessment, plaque specimens were taken. Subgingival specimens were only taken from the PT and PI individuals; buccal as well as supragingival samples were taken from the PH, PT, and PI patients. Applying a sterile micro-brush, buccal specimens were taken from the mucosa of each of the cheeks and put into a different sterile 1.5 mL microcentrifuge tube. In order to separate the supragingival specimen from any peripheral blood or saliva, the region has been dried by using a cotton roll prior to collecting the specimen. Utilizing a sterile Gracey curette, the subgingival specimens were taken from the peri-implant area and the deepest periodontal pocket. The specimens were then put in the tube that was previously indicated. Each sample was obtained and kept for further processing at -80°C.

## Whole genome DNA extraction:

A DNA purification kit was used to extract the samples' total DNA in accordance with the manufacturer's instructions. A Nano Drop ND-1000 spectrophotometer was used to measure the final concentrations, and the samples were kept at -80°C until needed. In this study, final concentrations were obtained for seven most common periodontal pathogens namely *Aggregatibacter actinomycetemcomitans*, *Fusobacterium nucleatum*, *Treponema denticola*, *Porphyromonas gingivalis*, *Prevotellaintermedia*, *Staphylococcus aureus* and *Haemophilus parainfluenzae*. The abundance of microorganisms was represented in the form of Log10CFU (x103).

## Statistical analysis:

SPSS version 21 was used for statistical analysis. For every collection of data, a number of normalcy tests were run. ANOVA was used to evaluate the mean clinical parameters, and P<0.05 was set as the statistical significance level for the post hoc Games-Howell test. After rare firing the operational taxonomic unit database, two measures were employed to assess alpha diversity: the Shannon index was utilized to quantify the homogeneity of the sample microbiota, and the Chao1 index was used to estimate species richness. To assess the significance of variations in the alpha diversity indices between the groups (P<0.05), the Mann-Whitney U test was run.

## Results:

The age of study participants was 56.8±11.2 years in patients with periimplantitis, 51.8±10.9 years in periodontitis and 27.0±6.8 years in normal healthy subjects. PPD calculated as mean of all sites was 3.3±0.5mm, 3.6±0.9mm and 2.5±0.05 mm in peri-implantitis category, periodontitis category and normal healthy subjects respectively. The PPD at subgingival sampled locations was 7.9±1.8mm in periimplantitis, 7.8±2.2mm in periodontitis patients. Similarly, the CAL was 3.8±0.8mm, 4.1±1.3mm, 2.5±0.05mm in peri-implantitis category, periodontitis category and normal healthy subjects respectively. The mean values of GI and PI was 0.7±0.3 and 0.5±0.04 in periimplantitis patients, 1.0±0.4 and 0.6±0.07 in periodontitis patients, 0.2±0.02 and 0.2±0.02 in normal subjects. The PPD, CAL, GI and PI were minimum in normal healthy subjects as compared to periimplantitis and periodontitis. The values of PPD, CAL, GI and PI were comparable in periodontitis and periimplantitis (Table 2).

On analysis of microbial profile of seven periodontal microbial pathogens it was observed that Log10CFU (x103) values of *Aggregatibacter actinomycetemcomitans*, *Fusobacterium nucleatum*, *Treponema denticola*, *Porphyromonas gingivalis* and *Haemophilus parainfluenzae* was maximum in patients diagnosed with periimplantitis while it was minimum in normal healthy subjects. The abundance of these micro-organisms in patients with periodontitis was greater than normal subjects but lesser than periimplantitis patients. On the other hand Log10CFU (x103) values of *Prevotella intermedia* and *Staphylococcus aureus* was greater in patients with periodontitis as compared to patients with periimplantitis and normal subjects. The values in periimplantitis were greater than normal subjects but lesser than chronic periodontitis for these microorganisms (Table 3).

## Discussion:

The most remarkable finding is that peri-implantitis is more common in people who have previously been diagnosed with periodontitis [14-18]. In particular, bacteria in the biofilm of teeth and implants release toxins that damage tissue, worsen the host's reaction to inflammation, and ultimately cause the nearby tissue to be destroyed. Inflammatory conditions PT and PI are associated with oral infection with polymicrobial species [19- 25]. However, the tissues surrounding dental implants are very different from the tissues surrounding teeth in a number of aspects, including the connective tissues shape, the absence of the periodontal ligament, and the underdeveloped vascular system. These differences raise the risk that PI will degrade and spread rapidly to support bone [26, 27, 28-29].The findings of our study are supported by some other studies. Previous studies have shown that the micro biota surrounding failed implants and periodontal damaged teeth create similar compositions, containing a sizable quantity of gram-negative anaerobic rods [21-26]. Along with the bacteria that are commonly thought of causing periodontal problems, some study investigations have revealed large quantities of *S. aureus* inside deeper peri-implant periodontal pockets that tend to be suppurative and exhibit bleeding following probing [22- 25]. The fact that titanium coatings are known to specifically attract *S. aureus* is noteworthy. Microbiological investigations have shown evidence of transmission from subgingival tooth regions to peri-implant habitats [19-24].Furthermore, research has shown that the subgingival biofilm composition of implant locations associated with healthy, normal dental implants is very different from that of pathogenic or failing implant sites [14-19].Implant-supported restorations are considered a standard treatment in modern prosthetic dentistry. Nonetheless, reports of biological as well as technical problems with dental implant rehabilitation have surfaced [16-21]. Biological problems associated with implants, such as peri-implant mucositis and periimplantitis, are often observed after a few years of use. Based on risk indicators and risk variables, the average incidence of peri-implantitis is thought to range from 8.9 to 43.3% at the implant location throughout a 10-year observation period [17-22].

Thanks to developments in prosthodontics and surgery, the average survival rate of durable dental implants is currently over 96% [6-13].But as the number of patients receiving implants has increased annually, so too has the frequency of peri-implant illnesses; currently, 45 percent of patients are thought to be affected. Periimplantitis (PI) is a degenerative peri-implant disease that is defined by an inflammatory infection in the peri-implant tissues and eventual loss of the bone that supports the implant, following initial bone remodeling [7- 12]. Lack of regular maintenance care, history of severe periodontitis, and insufficient brushing and flossing are risk factors for developing periodontitis (PT) [8-15]. There are some studies in which results obtained were not similar to our research. It has been noted that the microbiota found in the biofilms enclosing healthy implants is comparable to that seen in the tissues enclosing healthy teeth [20- 24]. Research revealed that the microbial makeup linked to chronic PT and peri-implant illness was comparable [21-25]. In our investigation, however, we discovered variations in the peri-pathogen abundance across the three groups of normal health, periodontitis, and periimplantitis [20-24].The aggressive and complex nature of PI may be the reason for the notable variations in microbiota composition observed in the PI group when compared to the PH and PT groups [12-17]. The NGS-identified microbial profile unique to PI may offer important information that is pertinent to the management of this illness [18- 23]. The establishment of an efficient and ideal treatment regimen for this illness will benefit from more investigation into the function of the special bacteria discovered in this study in relation to PI [21- 26].

## Conclusion:

The abundance of periopathogens evaluated in this research was different in periimplantitis patients, periodontitis patients and normal healthy subjects with slight more abundance of 75-80% of peripathogens in patient with periimplantitis.

## Figures and Tables

**Table 1:** Distribution of study participants

**Category**	**Participants**	**Number**
Category 1:	Periodontal healthy subjects (PH)	60
Category 2	Periodontitis (PT)	60
Category 3	Peri-implantitis (PI)	60

**Table 2:** Demographic details and data of periodontal parameters

	**Age (Mean±SD) years**	**Male : Female**	**PPD (mm) mean of all sites**	**PPD (mm) Subgingival sampled site**	**CAL (mm)**	**GI**	**Plaque index (PI)**
Peri-implantitis	56.8±11.2	01:01	3.3±0.5	7.9±1.8	3.8±0.8	0.7±0.3	0.5±0.04
Periodontitis	51.8±10.9	3.5:1	3.6±0.9	7.8±2.2	4.1±1.3	1.0±0.4	0.6±0.07
Normal healthy subjects	27.0±6.8	01:01	2.5±0.05	N/A	2.5±0.05	0.2±0.02	0.2±0.02

**Table 3:** Log10CFU (x103) of seven pathogenic periodontal pathogens at four clinical sites

	**Aggregatibacter actinomycetemcomitans**	**Fusobacterium nucleatum**	**Treponema denticola**	**Porphyromonas gingivalis**	**Prevotella intermedia**	**Staphylococcus aureus**	**Haemophilus parainfluenzae**
Peri-implantitis (Mean±SD)	1.42±0.03	3.51±0.02	2.02±0.03	2.78±0.09	0.07±0.009	3.57± 0.08	3.64±0.05
Periodontitis (Mean±SD)	1.37±0.08	3.06± 0.07	1.88	2.31± 0.02	0.18±0.09	3.71±0.02	3.40±0.01
Healthy subjects (Mean±SD)	1.26± 0.07	1.74± 0.05	1.27± 0.08	1.68±0.09	0.20±0.003	1.65± 0.06	1.12±0.03
P value	0.023*	0.014*	0.039*	0.041*	0.011*	0.015*	0.003*
